# Glutathione conjugation of sesquimustard: in vitro investigation of potential biomarkers

**DOI:** 10.1007/s00204-024-03788-1

**Published:** 2024-05-23

**Authors:** Muharrem Cenk, Havva Bekiroğlu Ataş, Suna Sabuncuoğlu

**Affiliations:** 1 Department of Toxicology, Faculty of Pharmacy, Hacettepe University, Ankara, Turkey; 2 General Directorate of Public Health, National Public Health Reference Laboratory, Ankara, Turkey

**Keywords:** Sesquimustard, Glutathione, HaCat, LC-HRMS, GSH conjugation

## Abstract

Sesquimustard (Q) is a powerful blistering agent that contains additional sulfur atoms. Sulfur mustard causes covalent bonding by alkylating nucleophilic groups of biologically important macromolecules such as lipids, proteins, DNA, or RNA. Most cells maintain relatively high amounts of a unique tripeptide called glutathione (GSH) (γ-glutamyl-cysteinyl glycine), which possesses a free thiol group, to prevent unwanted reactions caused by reactive chemical entities. Moreover, these thiol groups on cysteines (Cys) are the main target for alkylation. Although Q is the most potent vesicant among sulfur mustards, research studies identifying biomarkers of Q are very limited. Therefore, here in this study, we aimed to identify the GSH and Cys conjugates of Q using mass spectrometric methods and to observe the formation of these conjugates in HaCat cell culture following exposure to different doses. We identified four different conjugates of Q, which are bis-glutathionyl ethylthioethylthioethyl conjugate (GSH-ETETE-GSH), hydroxyethylthioethylthioethyl glutathione conjugate (HETETE-GSH), bis-cysteinyl ethylthioethylthioethyl conjugate (Cys-ETETE-Cys), and hydroxyethylthioethylthioethyl cysteine conjugate (HETETE-Cys). The identity of the conjugates was elucidated using liquid chromatography–high-resolution mass spectrometry (LC-HRMS). We also investigated changes in conjugate formation with exposure concentration and time elapsed after exposure in the cell culture. After exposure, GSH conjugates decreased until 1st hour, while Cys conjugates increased until 6th hour. We also observed that conjugate formation depended on the concentration of Q. This is the first study to elucidate the conjugates of Q dependent on GSH conjugation. As biomarkers are essential tools for evaluating exposure to Q, this study contributes to the limited number of studies identifying biomarkers for Q.

**Supplementary Information:**

The online version contains supplementary material available at 10.1007/s00204-024-03788-1.

## Introduction

**Fig. 1:**
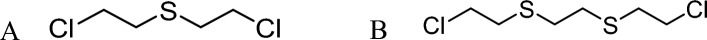
Chemical structure of **A** HD, **B** Q

Bis(2-chloroethyl sulfide) (Fig. 1A), also known as Sulfur Mustard or HD, was first used as a chemical warfare agent during World War I. After its first use, it was banned under the Geneva Protocol in 1925 and then under the Chemical Weapons Convention in 1993. The convention entered into force on 29th April 1997. However, it has been used in various conflicts and terrorist attacks throughout the past century, from World War I to the Syrian Civil War (Black 2016; McCauley 2020).

HD is a potent blistering agent that can cause severe damage to the eyes, skin, and respiratory system due to its vesicant properties. Chronic exposure to HD may also have carcinogenic and mutagenic effects (Dachir et al. 2019).

There are nine compounds, including HD, among the sulfur mustards in Schedule 1 of the CWC (Organisation for the Prohibition of Chemical Weapons, OPCW) (https://www.opcw.org/chemical-weapons-convention/annexes/annex-chemicals/schedule-1). Of these compounds, 1,2-bis(2-chloroethylthio)ethane (Sesquimustard) is the most potent vesicant known (Ellison 1999). It has been reported that the vesicant property of sesquimustard (Q) (Fig. 1B) is five times greater than HD (Gasson et al. 1948; Timperley et al. 2021). Q was first synthesised by Bennett and Whincop in 1921 (Bennett and Whincop 1921). During World War II, the United Kingdom developed a process that produced a mixture containing Q and HD. The mixture obtained by this process contained 70% HD and 30% Q. It has been reported that this mixture, called HQ, has better vesicant properties than HD (Timperley et al. 2003). It is also known that some of the HD stored in containers or ammunition converts to Q (Wagner et al. 1999).

Unlike other sulfur mustard compounds, Q is a solid at low temperatures (Mp: 56.6 °C) with much lower volatility and vapour pressure than HD (Hoenig 2007). These physico-chemical properties limit the effectiveness of Q as a CW agent. However, if it mixed with HD in certain proportions, the activity can be increased (Young and Bast 2020).

**Fig. 2:**
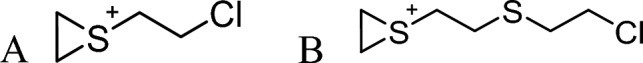
Episulfonium ion of **A** HD, **B** Q

In aqueous environments, HD forms a cyclic episulfonium ion, which is a highly reactive electrophile (Fig. 2A). Through this ion, HD reacts with biological molecules as a bifunctional alkylating agent, and it can react by binding directly to a molecule or by forming cross-links between two molecules (Jenner 2016). John et al. (2020) hypothesize that this process is the primary cause of HD toxicity and represents the fundamental chemical mechanism of cellular, tissue, and organ damage. St. Quintin et al. (2003) reported that Q transforms into three-membered ring episulfonium ions (Fig. 2B) and six-membered ring episulfonium ions in aqueous environments, similar to HD. However, due to the greater stability of six-membered ring episulfonium ions, the reaction occurs via three-membered ring episulfonium ions. In the same study, researchers also stated that this reaction is likely to take place in biological systems (St Quintin et al. 2003). The study by Blum et al. (2020) also reported that Q is conjugated to the nucleophilic Cys34 residue of human serum albumin (HSA) primarily via three-membered ring episulfonium ions.

HD causes covalent modifications by alkylating the nucleophilic groups of biologically important molecules such as proteins, DNA, RNA, or glutathione (GSH). In particular, thiol groups on cysteines are the main target for alkylation. Covalent modifications resulting from the reaction of HD with nucleophilic groups are characterized by the addition of a "hydroxyethylthioethyl" (HETE) moiety (Schmeißer et al. 2022). The metabolites resulting from these modifications are used to confirm exposure. Many methods have been developed to identify and determine metabolites of HD consisting of DNA, protein, and GSH conjugates in biomedical samples (Rybal'chenko et al. 2021; Witkiewicz and Neffe 2020; van der Schans 2020; Golime et al. 2019; Steinritz and Thiermann 2015; Xu et al. 2014). However, unlike HD, studies identifying biomarkers of Q are very limited. So far, only adducts formed by plasma proteins have been identified (Blum et al. 2020; Hemme et al. 2021; Chen et al. 2022). It can be understood from these studies that Q is metabolised in a similar way to HD. On the other hand, given that the majority of HD is conjugated to GSH prior to excretion (Etemad et al. 2019) and the majority of the detectable metabolites are the hydrolysis product thiodiglycol, and metabolites resulting from GSH conjugation (Jenner 2016), it would be an option to examine the conjugation of Q with GSH to determine Q exposure.

GSH conjugation begins with a reaction catalysed by glutathione-S-transferases (GSTs). Bifunctional electrophilic compounds such as HD undergo metabolic transformations that form mono- and bis(glutathionyl)S-conjugates. The resulting GSH-S conjugates are first converted to cysteinylglycine S-conjugates and then to cysteine (Cys) S-conjugates. Cys S-conjugates are finally N-acetylated to form *N*-acetyl-l-cysteine (NAC) S-conjugates, called mercapturic acid. Oxidation of the sulfur of NAC S-conjugates of HD to sulfoxides and sulfones is also possible in vivo (Cooper and Hanigan 2018). In a previous study, Cys and GSH conjugates were identified in the medium of HaCat cells exposed to the monofunctional sulfur mustard compound 2-chloroethyl ethyl sulfide (CEES) (Roser et al. 2021). Furthermore, bisglutathionyl and monoglutathionyl conjugates of HD were identified in microsomal and cytosolic fractions of human and pig liver exposed to HD by Halme et al. (2015). In this study, we aimed to identify the GSH and Cys conjugates of Q using mass spectrometric methods and to observe the formation of these conjugates in the human keratinocyte cell line (HaCat) following exposure to different doses. Ultimately, we report four different conjugates of Q, which are bis-glutathionyl conjugate (GSH-ETETE-GSH), mono-glutathionyl conjugate (HETETE-GSH), bis-cysteinyl conjugate (Cys-ETETE-Cys), and mono-cysteinyl conjugate (HETETE-Cys). To the best of our knowledge, this is the first study to elucidate the conjugation of Q with GSH.

## Materials and methods

### Chemicals

3,6-Dithia-1,8-octanediol (Q-glycol), thionyl chloride, l-Glutathione reduced, l-Cysteine hydrochloride, sodium bicarbonate, and dimethyl sulfoxide were purchased from Sigma-Aldrich (St. Louis, USA). Sodium hydroxide (NaOH), sodium sulfate and formic acid (FA) were purchased from Merck (Darmstadt, Germany). Dichloromethane (DCM) (Merck, Darmstadt, Germany) used for gas chromatography analysis was MS-SupraSolv grade. Liquid chromatography solvents, water, and acetonitrile (ACN) were Supelco (Bellefonte, USA) and were both LC–MS grade.

We prepared Q and isolated the conjugates from the mixture resulting from the reaction of Q with Cys and GSH.

*Caution: Q is a potent vesicant. It should only be handled in a properly functioning fume hood by experienced and well-trained personnel using suitable protective equipment. Thorough decontamination of all materials in contact with the toxin is also required.*

### Preparation of Q

Q was synthesized by modifying a previously published procedure (Reeves et al. 1955). Its purity, determined by a gas chromatography-mass spectrometer combined with a dual flame photometric detector (GC–MS/dFPD), was found to be above 95%.

### Direct reaction of GSH and Cys with Q

The solution of Q (0.1 mmol in 10 mL ACN) was added dropwise to the solution of GSH (0.2 mmol in 10 mL saturated sodium bicarbonate). The mixture pH was adjusted to 8–9 with 0.1 M NaOH. The reaction mixture was stirred at room temperature, and the reaction progress was monitored using GC–MS/dFPD. The reaction continued until Q was consumed. The mixture was rinsed with DCM (3 × 5 mL), and the aqueous layer was evaporated using reduced pressure. The reaction between Q and Cys was performed using the same method and conditions described in Bielmann et al.'s (2018) study. The reaction end products were isolated from the reaction mixtures using a Shimadzu HPLC system (Kyoto, Japan). The liquid chromatography system comprised a Nexera X2 LC-30AD pump, Nexera X2 SIL-30AC autosampler, SPD-M40 photodiode array detector (PAD), CTO-10AS VP column oven, FRC-10A fraction collector, and a C18 reverse-phase column (ACE 5, C18, 5 µm, 4.6 × 250 mm). The following solvent gradient of mobile phase A (0.1% FA) and mobile phase B (ACN, 0.1% v/v FA) was applied at a flow rate of 1 mL/min for liquid chromatography: t [min]/B [%]: 0/0; 10/5; 30/20; 45/30; 50/30; 50.1/0. The total analysis time, including a 4.9-min equilibration time, was 55 min. The column temperature was kept at 60 °C throughout the analysis. The reaction products were collected in approximately 2 mL fractions at 210 nm. These fractions were used for identification in LC-HRMS and multiple reaction monitoring mode (MRM) optimizations in liquid chromatography-tandem mass spectrometry (LC–MS/MS).

### In vitro experiments

#### Cell culture

The skin serves as a direct target organ for blister agents such as Q. Therefore, experiments were conducted using a human keratinocyte cell line, HaCaT cells (Thermo Fisher Scientific, Grand Island, USA), representing the main cell type in the epidermal layer of human skin. HaCaT human keratinocytes were cultured in Dulbecco's Modified Eagle Medium (DMEM) (Biowest, France), including 10% (v/v) fetal calf serum and 1% (v/v) antibiotic mixture (50 U/mL penicillin and 50 mg/mL streptomycin). The cells seeded in 75 cm^2^ cell culture flasks were grown in an incubator (Heraeus, Hanau, Germany) at 37 °C under conditions of 5% CO_2_.

#### Treatments of the cells

To facilitate the application, each 75 cm^2^ flask was seeded with 4 × 10^6^ cells and incubated for 24 h to allow for adherence. Prior to application, the medium was removed. Q solution prepared in Dulbecco's Phosphate Buffered Saline (DPBS) (Biowest, Nuaillé, France) at different final concentrations (0 µM (control), 25 µM, 50 µM, 100 µM, and 250 µM) was added to the cells and incubated at room temperature for 30 min. After incubation at room temperature, DPBS was collected for analysis. The cells were then washed twice with DPBS to eliminate Q residues. Subsequently, the cells were incubated with fresh medium at 37 °C for 6 h. Medium samples were collected at different time points (0 min, 10 min, 1 h, 3 h, and 6 h). Collected samples were stored at -80 °C until the sample preparation process.

After 6 h of incubation, the medium was discarded, and the cells were incubated with 0.25% trypsin and collected in a centrifuge tube. The cells were centrifuged at 1000 rpm for 5 min (Hettich, Tuttlingen, Germany), and the supernatant was removed. The cell pellets were suspended in 1 mL of DPBS, centrifuged (500 rpm, 5 min), and the supernatant was eliminated again. The cell pellets were stored at -80 °C until further analysis.

Additionally, an application was performed to observe the products formed during the application of Q at the same concentrations in DPBS for only 30 min. The cells were treated with Q at room temperature for 30 min in DPBS. After that, the same procedures were applied to the other groups of cells, and the cells were removed from the flasks and stored at -80 °C until sample preparation.

Experiments were conducted three times for each concentration.

### Sample preparation

The samples, collected from DPBS and medium, were initially centrifuged at 10,000 rpm for 10 min using a Hettich Mikro 22R centrifuge. Then, they were filtered through 0.2 μm PTFE filters (Millex, Cork, Ireland), diluted by half with 0.1% FA, and analyzed by LC–MS/MS.

For the cell lysate preparation, 500 µL of cold methanol was added to the cell pellets and vortexed for 5 min. After approximately 15 min at room temperature, the mixture was centrifuged at 10,000 × rpm for 10 min. The supernatant was collected and evaporated to dryness under nitrogen. It was then reconstituted in 100 µL of 0.1% FA, filtered through 0.2 μm PTFE filters, and analyzed by LC–MS/MS.

### LC-HRMS analysis

The products obtained from the reaction of Q with GSH and Cys were identified using an LC-Q-TOF/MS system. The system consisted of an autosampler, a column oven, and a binary pump in a 1290 Infinity II UHPLC (Agilent Technologies, Santa Clara, USA) system. A C18 column (2 μm, 2.1 × 100 mm, Merck Purospher STAR RP-18 end-capped, Darmstadt, Germany) was used during the analysis. For liquid chromatography, the following solvent gradient of mobile phase A (0.1% FA) and mobile phase B (ACN, 0.1% v/v FA) was applied at a flow rate of 0.4 mL/min: t [min]/B [%]: 0/5; 2/10; 3/10; 4/20; 5/20; 6/35; 7/35; 8/70; 9/70; 9.01/5. The total analysis time, including a 1.99-min equilibration time, was 11 min. The autosampler temperature was set at 15 °C, the column oven was maintained at 50 °C, and the injection volume was adjusted to 1 μL. To prevent contamination of the ion source, the initial 30 s of eluent flow were directed to waste. Mass spectrometry was conducted using an Agilent Jet Stream dual electrospray ionization source (AJS ESI) coupled to a 6550 iFunnel Accurate-Mass Q-TOF/MS system (Agilent Technologies, Santa Clara, USA). Ionization was carried out in positive mode, and the operational parameters were as follows: voltages of capillary, nozzle, fragmentor and octopole 1 RF were set to 3500 V, 800 V, 80 V, 750 V respectively. Drying gas and sheath gas flows were set 12 L/min. In addition, the nebuliser pressure was 35 psig and the sheath gas temperature was 300 °C. Full scan MS data were acquired at a rate of 5 spectra per second in the m/z range of 50–950 at 2 GHz. For MS data acquisition and processing, Agilent MassHunter Data Acquisition (B.10.00) and Agilent MassHunter Qualitative Analysis (B.10.00) were used, respectively. MS/MS experiments were conducted with approximately 4 amu medium isolation width and collision energies ranging from 5 to 40 eV for all compounds. The instrument underwent daily calibration using the manufacturer's calibration solution before analysis. Purine (m/z 121.0509) and HP-921 (m/z 922.0098) were used as internal reference for mass corrections.

### LC–MS/MS analysis

The conjugates in the cells and the medium samples were analyzed using a Shimadzu (Kyoto, Japan) LC–MS 8060 triple quadrupole mass spectrometer equipped with a heated ion source operating in positive ionization mode. The system comprises an LC-30AD pump, a SIL-30AC autosampler, and a CTO-10AS VP column oven. Data were collected in multiple reaction monitoring (MRM) scanning mode. The column and the mobile phase gradient program used in these analyses were the same as those used in the LC-HRMS system. Optimized ionization parameters were as follows: Nebulizing gas flow: 3 L/min; heating gas flow: 10 L/min; drying gas flow: 10 L/min; interface temperature: 300 °C; desolvation line temperature: 525 °C; heat block temperature: 400 °C; DL temperature: 250 °C. The unit mass resolution was used to monitor mass transitions. Other parameters associated with the compounds are listed in Table 1. Data acquisition and evaluation processes were performed using Lab Solutions LCMS Ver.5.97 (Shimadzu, Kyoto, Japan).

**Table 1:** Optimized MS/MS parameters for conjugates

Analytes	MRM Transition m/z (Q1 → Q3)	Q1 (V)	CE (V)	Q3 (V)	Dwell Time (msec)
HETETE-Cys	286 → 120	– 14	– 21	– 23	54
	286 → 105	– 14	– 21	– 23	54
	286 → 148	– 14	– 21	– 23	54
Cys-ETETE-Cys	389 → 120	– 19	– 27	– 20	82
	389 → 148	– 19	– 19	– 10	82
	389 → 180	– 20	– 25	– 30	82
HETETE-GSH	472 → 105	– 13	– 33	– 19	52
	472 → 177	– 23	– 30	– 18	52
	472 → 197	– 23	– 25	– 20	52
GSH-ETETE-GSH	761 → 503	– 22	– 47	– 19	52
	761 → 632	– 22	– 29	– 24	52
	761 → 177	– 22	– 21	– 24	52

CE: Collision energy

## Results and discussion

Identification and confirmation of exposure and elucidation of the substance's toxicity are crucial in guiding the treatment of casualties and establishing evidence of CWC violations. Therefore, it is essential to identify reliable and discriminative biomarkers in the retrospective analysis of chemical warfare agent exposure for forensic identification and clinical diagnosis. In this study, potential biomarkers resulting from GSH conjugation that may allow the confirmation of Q exposure were investigated.

This study is divided into two parts. The first part involves identifying and characterizing potential biomarker candidate conjugates arising from the reaction of Q with GSH and Cys using LC/HRMS. The second part of the study observes the formation of GSH conjugates in the HaCat cell culture due to exposure to Q and the subsequent transformation of these conjugates into Cys conjugates. Additionally, this study investigates changes in conjugate formation with exposure concentration and time elapsed after exposure in the cell culture.

### Identification of conjugates by LC-HRMS

To determine the identity of the reaction products of GSH and Cys with Q, their accurate masses, elemental compositions, and isotopic patterns were determined first using LC-HRMS. Additionally, the structures of the product ions generated during collision-induced dissociation (CID) were elucidated. The obtained accurate masses, elemental compositions, and isotopic patterns were compared with the theoretically predicted ones and were confirmed. All the obtained results facilitated the selective identification of candidate compounds and provided unambiguous elucidation of their structures. Table 2 presents the mass errors in ppm and mDa, which were calculated by comparing the measured masses of precursor ions with their exact isotopic masses, along with the corresponding molecular structures. Isotopic distributions for all conjugates are given in supporting information in Fig. SI–SI 4 and Table SI 1–SI 4.

**Table 2:**
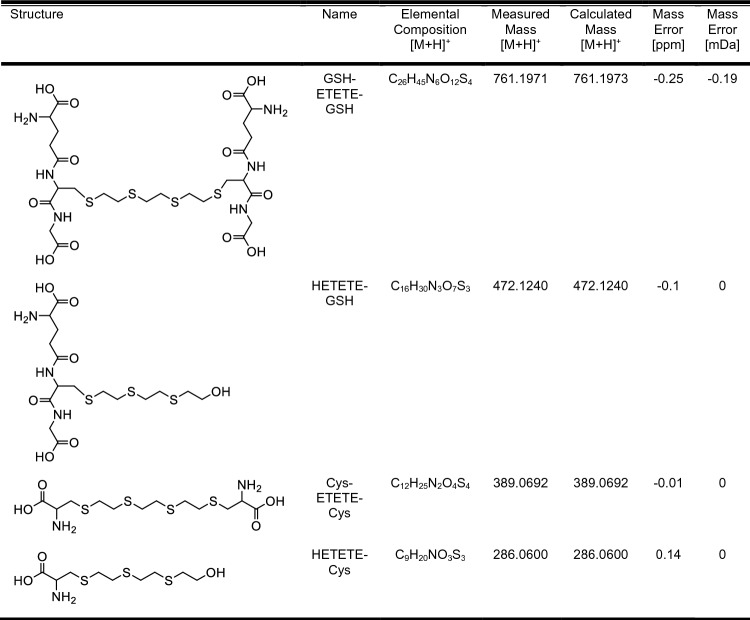
Molecular structures and calculated mass errors of conjugates

**Fig. 3:**
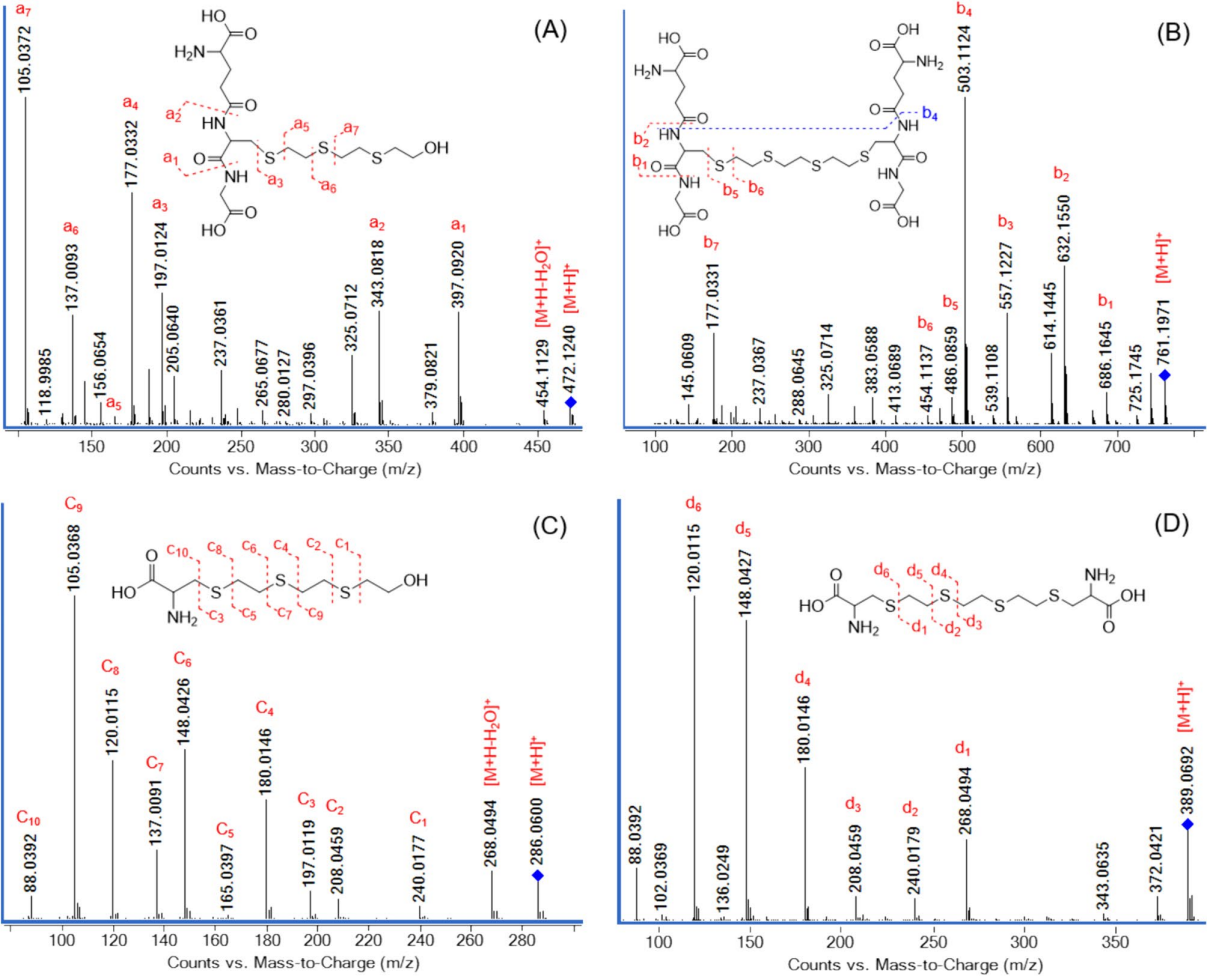
Product ion spectra of the conjugates of Q with GSH and Cys. The products resulting from the reaction of Q with GSH and Cys were isolated from the reaction mixture by HPLC-FRC and analyzed by LC-Q-TOF in Auto-MS/MS mode. The spectra extracted from the chromatographic peaks were labeled with the most prominent and specific product ions obtained from single proton precursor ions [M + H]^+^. **A** HETETE-GSH (20 CE), **B** GSH-ETETE-GSH (30 CE), **C** HETETE-Cys (12 CE), **D** Cys-ETETE-Cys (18 CE)

To verify the structures of conjugated candidate molecules, high-resolution product ion spectra (PIS) were utilized to characterize fragmentation patterns. The structures of the resulting fragment ions were determined (Fig. 3).

During CID, most GSH conjugates produce specific neutral losses and fragment ions. The product ions resulting from neutral losses of glycine (m/z 75.0320) and pyroglutamic acid (m/z 129.0426) serve as primary evidence for the identification and confirmation of a GSH conjugate (Wen and Fitch 2009). In addition, product ions resulting from neutral losses of γ-glutamyl-alanyl-glycine (glu-ala-glc) (m/z 275.1117) and GSH (m/z 307.0838) strengthen this evidence. Product ion containing residue of cysteinylglycine (m/z 177.0328) is frequently observed in the fragmentation of GSH conjugates (Murphy et al. 1992; Brink et al. 2014). Neutral loss of cysteine (m/z 121.0197) is also a common and specific neutral loss observed during CID of cysteine conjugates (Ma et al. 2014).

As it is shown in Fig. 3A, B, the a_1_ and b_1_ ions correspond to ions resulting from the neutral loss of glycine, while the a_2_ and b_2_ ions arise from the neutral loss of pyroglutamic acid. Additionally, the a_3_ and b_5_ ions correspond to glu-ala-glc, and the a_5_ (m/z 165.1403) and b_6_ ions correspond to neutral loss of GSH. Both conjugates show a specific fragmentation product ion originating from cysteinylglycine and are labeled as a_4_ and b_7_ on the spectra. Furthermore, GSH-ETETE-GSH exhibits multiple neutral losses. The b_4_ ion results from the neutral loss of two pyroglutamic acids, and the b_3_ ion results from the neutral loss of one glycine and one pyroglutamic acid. GSH-ETETE-GSH and HETETE-GSH generally demonstrate the typical fragmentation pattern of GSH conjugates. The obtained results were consistent with previous research on the examination of GSH conjugates of nitrogen mustard and HD, demonstrating similar fragmentation patterns (Halme et al. 2015; Hamzah et al. 2023).

Cysteine neutral loss (m/z 121.0197) is a common and specific neutral loss observed during CID of cysteine conjugates (Ma et al. 2014). The c_5_ and d_1_ ions in the product ion spectra of the Cys conjugates of Q in Fig. 3C, D, correspond to ions formed as a result of neutral loss of cysteine. Compared to GSH conjugates, the fragmentation pattern of cysteine conjugates is relatively simpler. Moreover, it seems that the cleavage reaction mainly occurs due to the cleavage of the C-S bond.

In addition, the HETETE-GSH and HETETE-Cys monoconjugates contain characteristic ions previously reported by CID of some protein adducts of HD and Q (Blum et al. 2020; Hemme et al. 2021; John et al. 2019; Braun et al. 2017; Steinritz et al 2021). These ions are associated with the thioalkyl chain of the conjugates. In Fig. 3A and Fig. 3C, the a_3_ and c_3_ ions correspond to the HETETE-S moiety, along with the sulfur atom of Cys. The a_5_ and c_5_ ions represent the HETETE moiety. The a_6_ and c_7_ ions correspond to the HETE-S moiety, along with the adjacent sulfur atom, while the a_7_ and c_9_ ions correspond to the HETE moiety.

Tables SI 5 to SI 8 provide detailed information on the structural assignment, elemental compositions, theoretical and measured masses, and mass errors of each ion of the conjugates. After evaluating all the results, we have ensured the selective identification of candidate compounds and elucidated their structures without any ambiguity.

### Detection of GSH and Cys conjugates of Q in HaCat cell culture

**Fig. 4:**
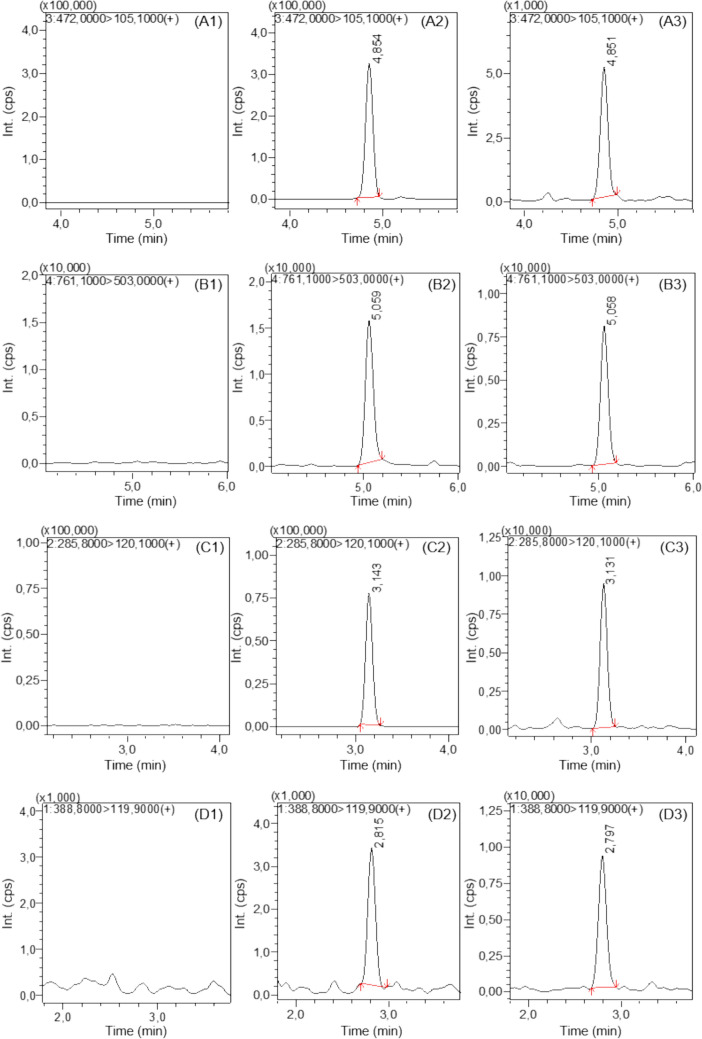
Detection of GSH and Cys conjugates in cells exposed to 250 µM Q for 30 min. LC–MS/MS (MRM) chromatograms of **A** HETETE-GSH, **B** GSH-ETETE-GSH, **C** HETETE-Cys, **D** Cys-ETETE-Cys in control (1), sample (2) and reference (3)

The products isolated from the reaction mixture of Q with GSH and Cys enabled method optimization in MRM mode using LC–MS/MS. In the optimized method, specific and most abundant fragments of the molecular ions of the analytes were monitored in ESI mode. Reporting criteria in the OPCW guidelines for LC–MS/MS results were used during the evaluation of the results (OPCW 2023). The background noise was low, and none of the conjugates were detected in the control samples. All conjugates were detected in cells exposed to Q for 30 min (Fig. 4). On the other hand, only cysteine conjugates (HETETE-Cys and Cys-ETETE-Cys) were observed in the cell lysates at 6th hour. While GSH-ETETE-GSH was not detected in medium samples, Cys-HETETE was the only conjugate detectable in all doses and time points. GSH-HETETE was only detected in the medium at 0, 10 min, and 1 h time points after 250 µM Q exposure. Also, Cys-ETETE-Cys was detected in the medium at 1 h, 3 h, and 6 h time points after the concentrations of 100 µM and 250 µM Q exposure.

**Fig. 5:**
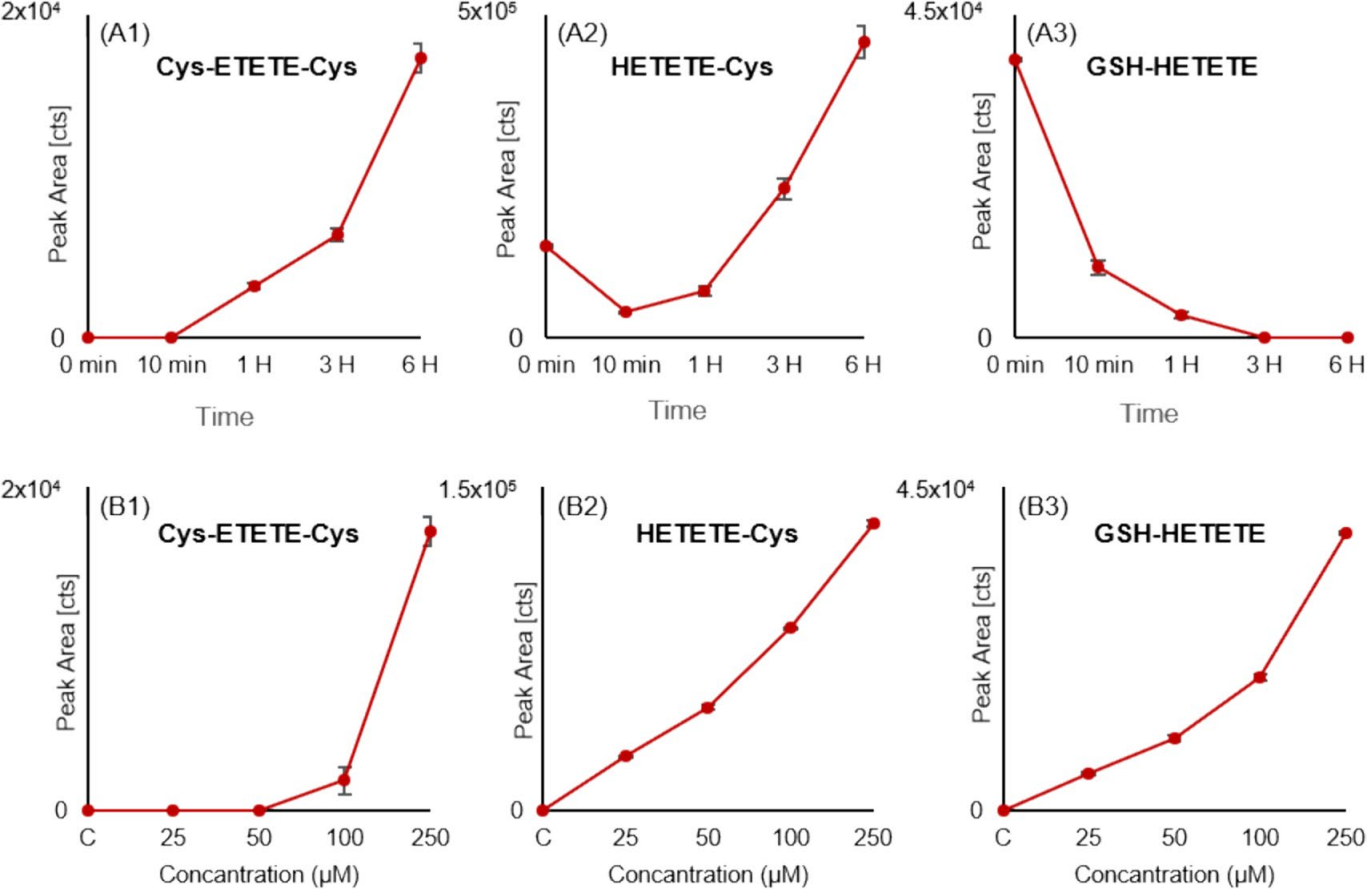
Changes in conjugate formation with concentration and time elapsed after exposure **A** time-dependent variation of conjugate formation with 250 µM Q exposure; **B** concentration-dependent variation of conjugate formation at 0 min (B1) and (B2), 6th hour (B3). C: Control, H: Hour

The analysis results of the samples obtained from HaCat cell culture studies showed a decrease in GSH conjugates over time in both cells and the medium, while Cys conjugates were found to be more persistent (Fig. 5A). Furthermore, it was observed that conjugate formation was dependent on the concentration of Q (Fig. 5B). This change in conjugates can be explained by the primary metabolism of GSH conjugates to Cys conjugates in the mercapturic acid metabolism similar to the literature data (Hanna and Anders 2019).

This is an important biotransformation pathway for toxic electrophilic substances, begins with the GSH conjugation catalyzed by GST enzymes (Tierbach et al. 2020). GSTs play a physiologically significant role in the detoxification of potential alkylating agents by catalyzing the reaction of electrophilic compounds through the sulfhydryl group of GSH. Electrophiles entered to the cell are converted to GSH-S conjugates via GSTs and transported to the cell surface by protein carriers (MRP1). Here, the ɣ-glutamyl moiety is cleaved by ɣ-glutamyl transferase (ɣ-GT) and the glycine residue by dipeptidase, respectively, and converted into the cysteinylglycine S-conjugate. Both ɣ-GT and dipeptidases are membrane-bound enzymes. Therefore, biotransformation occurs extracellularly after transporting the glutathione conjugate out of the cell, and then the cysteine conjugate is retransported into the cell (Hinchman and Ballatori 1994). In this case, GSH and Cys conjugates can be expected to be observed in both medium and cell lysates. Our study results indicate the presence of both Cys and GSH conjugates in both the culture medium and the cells, which is consistent with the expected outcome of this process. However, GSH-ETETE-GSH was relatively lower than other conjugates within the cell and was likely either below the detection limit in the medium or metabolized very rapidly.

Mercapturic acid metabolism continues with the conversion of the cysteine conjugate re-transported to the cell into NAC conjugate by N-acetyltransferase (NAT) (Frigerio et al. 2019). However, the acetylation of cysteine conjugates is not a reaction that occurs ubiquitously in the biological system. This is because NAT enzymes are mostly expressed in renal proximal tubular cells and, to a lesser extent, in the liver. As a consequence, it has been reported that Cys-S conjugates cannot be converted to NAC-S conjugates in cultured skin cells and accumulate without being metabolized (Roser et al. 2021). Cys and GSH conjugates were detected in HaCaT cell culture exposed to CEES. However, NAC conjugate was not detected in this in vitro study. Furthermore, it was observed that the amounts of GSH-CEES and Cys-CEES detected in the medium were dose-dependent, and the concentration of Cys-CEES was higher than that of GSH-CEES at all time points. These results are consistent with the obtained data from our study. In the same study, plasma samples from SKH-1 mice exposed to CEES were analyzed one day after the exposure, and GSH-CEES, Cys-CEES, and NAC-CEES were detected. However, it is reported that the concentrations of GSH-CEES and Cys-CEES are much lower than NAC-CEES.

Gilardoni et al. (2021) conducted a study in which hairless SKH-1 mice were dermally exposed to CEES. Plasma samples were collected after the exposure, and the results of the analyses showed the presence of GSH-CEES, Cys-CEES, and NAC-CEES metabolites. The highest concentration of metabolites was determined one day after the exposure, and the concentrations decreased over time. However, it was reported that all three metabolites were still detectable at measurable levels after 14 days. The detected primary metabolites were Cys and NAC metabolites, with the concentration of GSH-CEES being much lower than the others.

Q and CEES are compounds with comparable chemical and toxicological properties. Consequently, it is hypothesized that the GSH and Cys conjugates found in cell culture after Q agent exposure may likewise form subsequent to in vivo exposure. Furthermore, these conjugates could serve as biomarkers of Q exposure and warrant extensive studies in this regard.

## Conclusion

In this study, we identified for the first time GSH and Cys conjugates associated with Q's GSH conjugation by using mass spectrometric method. Furthermore, we reported that the formation of these conjugates in HaCat cell culture varies in dose- and time-dependent manners. We propose these four specific conjugates, termed GSH-ETETE-GSH, HETETE-GSH, Cys-ETETE-Cys, and HETETE-Cys, as potential candidate exposure biomarkers for Q.

On the other hand, the obtained results shed light on further investigations of biotransformation products of Q. Previous studies have reported that Cys conjugates of HD are acetylated to NAC conjugates, NAC conjugates are oxidized to sulfoxides and sulfones (Cooper and Hanigan 2018), or converted to β-lyase metabolites via β-lyase enzymes (Read and Black 2004). Our future studies will also focus on the further metabolism of Cys metabolites resulting from GSH conjugation of Q, as they may undergo similar metabolic pathways. This study contributes to a limited number of studies identifying biomarkers for Q.

### Supplementary Information

Below is the link to the electronic supplementary material.

Supplementary file1 (PDF 760 KB)

## Data Availability

Data will be made available on request.

## Abbreviations

ACN: Acetonitrile
CE: Collision energy
CEES: 2-Chloroethyl ethyl sulfide
CID: Collision-induced dissociation
Cys: Cysteine
DMEM: Dulbecco's Modified Eagle Medium
DPBS: Dulbecco's Phosphate Buffered Saline
ESI: Electrospray ionization
ETETE: Ethylthioethylthioethyl linker
FA: Formic acid
FRC: Fraction collector
GC–MS/dFPD: Gas chromatography–mass spectrometer/ dual flame photometric detector
ɣ-GT: ɣ-Glutamyltransferase
glu-ala-glc: γ-Glutamyl-alanyl-glycine
GSH: Glutathione
GST: Glutathione S-transferases
HD: Bis(2-chloroethyl sulfide)
HETE: Hydroxyethylthioethyl moiety
HETETE: Hydroxyethylthioethylthioethyl moiety
HPLC: High-performance liquid chromatography
LC-HRMS: Liquid chromatography/high resolution mass spectrometry
LC–MS/MS: Liquid chromatography–tandem mass spectrometry
LC-Q-TOF/MS: Liquid chromatography quadrapol time-of-flight mass spectrometry
Mp: Melting Point
MRM: Multiple reaction monitoring mode
NAC: *N*-Acetyl-l-cysteine
NAT: N-acetyltransferase
PAD: Photodiode array detector
PBS: Phosphate Buffered Saline
Q: Sesquimustard
