# Supplementary material for: Glutathione conjugation of sesquimustard: in vitro investigation of potential biomarkers
Source: Arch Toxicol. 2024 May 23;98(9):2867–77. doi: 10.1007/s00204-024-03788-1 (PMC11324776; doi:10.1007/s00204-024-03788-1)
Supplement: Supplementary file 1 — Supplementary file1 (PDF 760 KB) [file 204_2024_3788_MOESM1_ESM.pdf]

Supplementary Information for Archives of Toxicology

Glutathione Conjugation of Sesquimustard: In vitro Investigation of Potential Biomarkers

Muharrem Cenk[1,2], Havva Bekiroğlu Ataş[2], Suna Sabuncuoğlu[1*]

[1] Department of Toxicology, Faculty of Pharmacy, Hacettepe University, Ankara, Türkiye
[2] General Directorate of Public Health, National Public Health Reference Laboratory, Ankara, Türkiye

[*]Corresponding author
Prof. Dr. Suna Sabuncuoğlu
Postal Address: Department of Toxicology, Faculty of Pharmacy, Hacettepe University, Ankara, Türkiye
ORCID: 0000-0002-9702-4214
e-mail: suna@hacettepe.edu.tr; sunaatasayar@gmail.com

Content

Figures

Figure SI 1. Extracted ion chromatogram (m/z 286.0600) in full scan mode and mass spectra of HETETE-Cys

Figure SI 2. Extracted ion chromatogram (m/z 389.0692) in full scan mode and mass spectra of Cys-ETETE-Cys.

Figure SI 3. Extracted ion chromatogram (m/z 472.1240) in full scan mode and mass spectra of HETETE-GSH

Figure SI 4. Extracted ion chromatogram (m/z 761.1973) in full scan mode and mass spectra of GSH-ETETE-GSH.

Tables

Table SI 1. The measured and the calculated isotope distribution of HETETE-Cys

Table SI 2. The measured and the calculated isotope distribution of Cys-ETETE-Cys

Table SI 3. The measured and the calculated isotope distribution of HETETE-GSH

Table SI 4. The measured and the calculated isotope distribution of GSH-ETETE-GSH

Table SI 5 Product ions of single protonated HETETE-GSH

Table SI 6 Product ions of single protonated GSH-ETETE-GSH

Table SI 7 Product ions of single protonated HETETE-Cys

Table SI 8 Product ions of single protonated Cys-ETETE-Cys

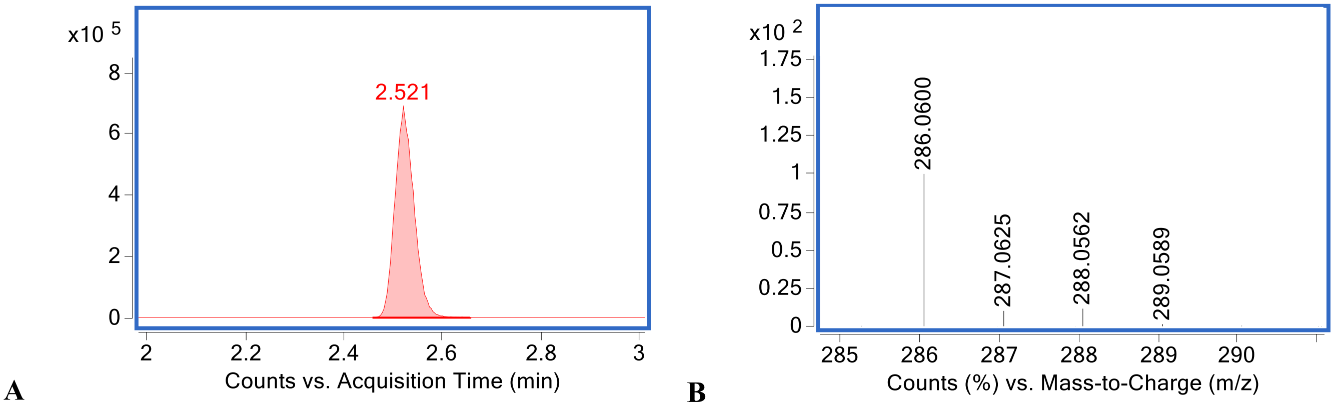

Fig. SI 1 A Extracted ion chromatogram (m/z 286.0600) in full scan mode and **B** mass spectra of HETETE-Cys

Table SI 1 The measured and the calculated isotope distribution of HETETE-Cys

m/z (Calc)	m/z	Height % (Calc)	Height %	Diff (ppm)	Diff (mDa)
286.0600	286.0600	100	100	0.14	0
287.0625	287.0625	12.81	10.07	-0.01	0
288.0566	288.0562	14.8	11.54	-1.52	-0.4
289.0590	289.0589	1.72	1.34	-0.35	-0.1

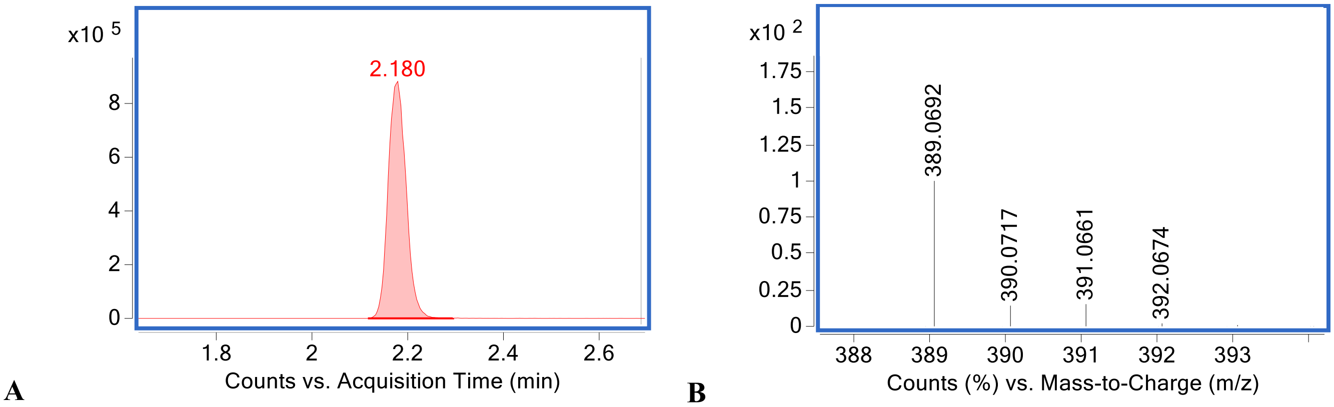

Fig. SI 2 A Extracted ion chromatogram (m/z 389.0692) in full scan mode and **B** mass spectra of Cys-ETETE-Cys.

Table SI 2 The measured and the calculated isotope distribution of Cys-ETETE-Cys

m/z (Calc)	m/z	Height % (Calc)	Height %	Diff (ppm)	Diff (mDa)
389.0692	389.0692	100	100	-0.01	0
390.0716	390.0717	17.31	14.23	0.27	0.1
391.0659	391.0661	20.13	15.25	0.45	0.2
392.0681	392.674	3.17	2.04	-1.76	0.7

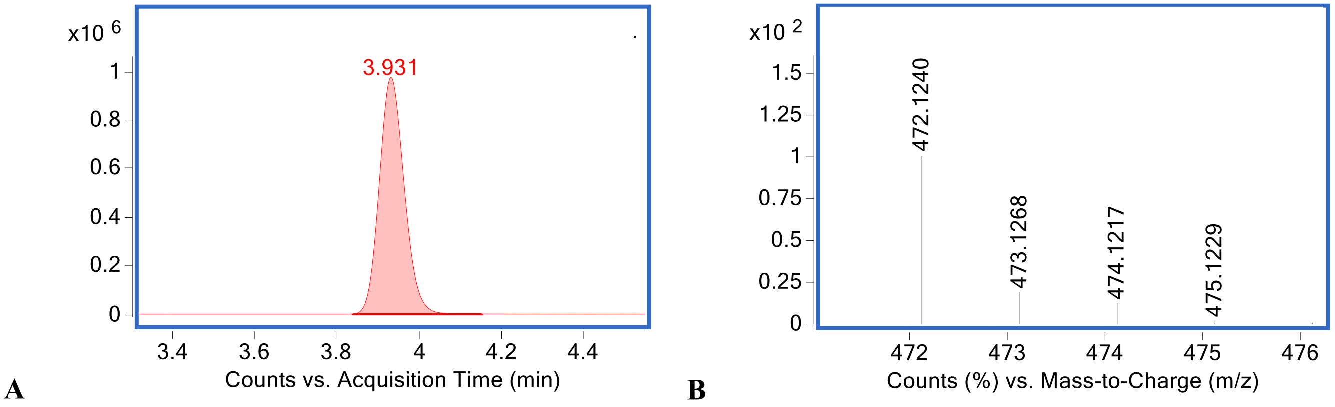

Fig. SI 3 A Extracted ion chromatogram (m/z 472.1240) in full scan mode and **B** mass spectra of HETETE-GSH

Table SI 3 The measured and the calculated isotope distribution of HETETE-GSH

m/z (Calc)	m/z	Height % (Calc)	Height %	Diff (ppm)	Diff (mDa)
472.1240	472.1240	100	100	-0.1	0
472.1267	473.1268	21.38	19	0.19	0.1
474.1218	474.1217	17.04	12.49	-0.17	-0.1
475.1238	475.1229	3.21	2.06	-1.93	-0.9

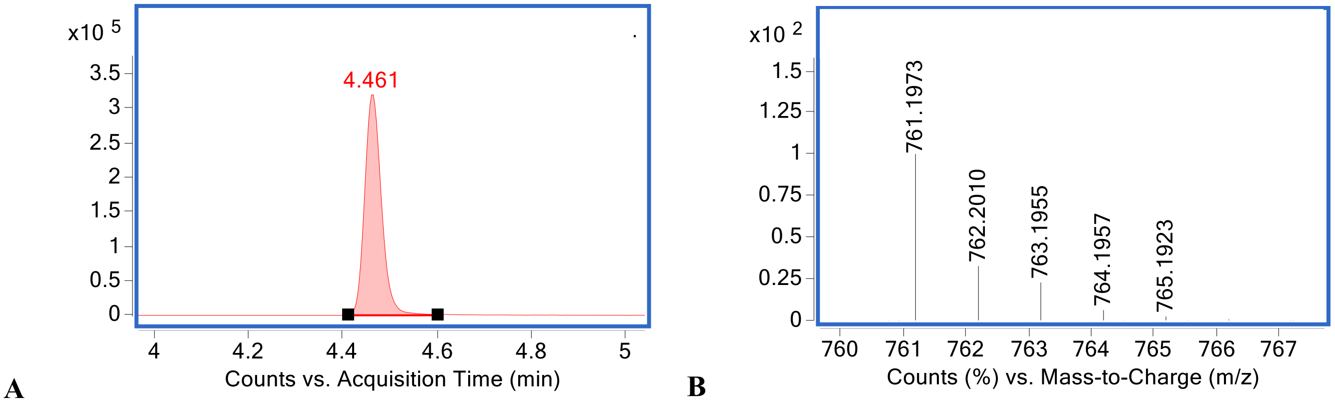

Fig. SI 4 A Extracted ion chromatogram (m/z 761.1973) in full scan mode and **B** mass spectra of GSH-ETETE-GSH.

Table SI 4 The measured and the calculated isotope distribution of GSH-ETETE-GSH

m/z (Calc)	m/z	Height % (Calc)	Height %	Diff (ppm)	Diff (mDa)
761.1973	761.1973	100	100	0.09	0.1
762.1999	762.2010	34.45	32.65	1.43	1.1
763.1960	763.1955	26.13	22.73	-0.59	-0.5
764.1975	764.1957	7.5	6.15	-2.44	-1.9
765.1949	765.1923	2.89	2.33	3.40	2.6

Table SI 5 Product ions of single protonated HETETE-GSH

**Structure**	**Elemental composition**	**Mass (Measured)**	**Mass (Calculated)**	Mass Error **[ppm]**	Mass Error **[mDa]**
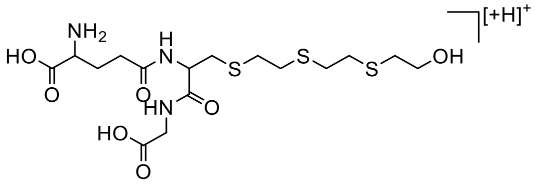	$C_{16}H_{30}N_3O_7S_3$	472.1240	472.1240	-0.19	-0.09
**$[M+H-H_2O]^+$**	$C_{16}H_{28}N_3O_6S_3$	454.1129	454.1135	-1.35	-0.61
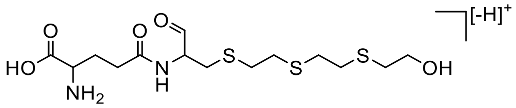<br>a_1= neutral loss of glycine from **[M+H]**$^+$	$C_{14}H_{25}N_2O_5S_3$	397.0920	397.0920	-0.03	-0.01
**$[a_1-H_2O]^+$**	$C_{14}H_{23}N_2O_4S_3$	379.0821	379.0815	1.72	0.65
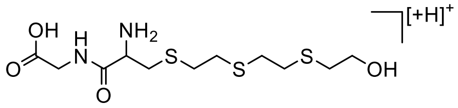<br>a_2= neutral loss of pyroglutamic acid from **[M+H]**$^+$	$C_{11}H_{23}N_2O_4S_3$	343.0818	343.0815	1.13	0.39
**$[a_2-H_2O]^+$**	$C_{11}H_{21}N_2O_3S_3$	325.0712	325.0709	0.94	0.31
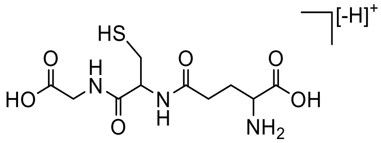	$C_{10}H_{16}N_3O_6S$	306.0749	306.0754	-1.88	-0.57
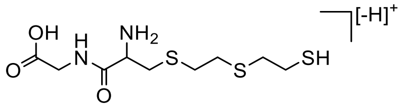	$C_9H_{17}N_2O_3S_3$	297.0396	297.0396	0.12	0.04
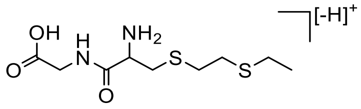	$C_9H_{17}N_2O_3S_2$	265.0677	265.0675	0.57	0.15
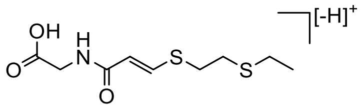	$C_8H_{14}N_3O_4S$	248.0406	248.0410	-1.27	-0.32

Table SI 5 *(continued)*

**Structure**	**Elemental composition**	**Mass (Measured)**	**Mass (Calculated)**	Mass Error **[ppm]**	Mass Error **[mDa]**
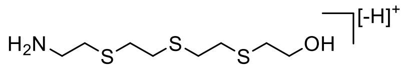	$C_8H_{18}NOS_3$	240.0548	240.0545	1.44	0.35
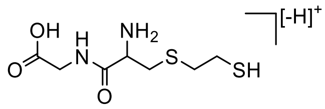	$C_7H_{13}N_2O_3S_2$	237.0361	237.0362	-0.40	-0.10
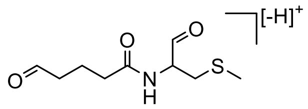	$C_9H_{14}NO_3S$	216.0684	216.0689	-2.31	-0.50
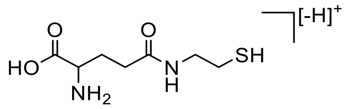	$C_7H_{13}N_2O_3S$	205.0640	205.0641	-0.77	-0.16
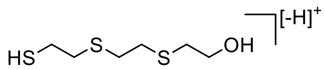<br>a_3= HETETE-S=neutral loss of ɣ-glu-ala-glc from **[M+H]**$^+$	$C_6H_{13}OS_3$	197.0124	197.0123	0.35	0.07
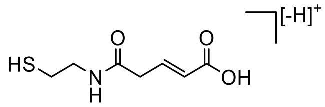	$C_7H_{10}NO_3S$	188.0378	188.0376	1.24	0.23
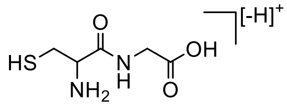<br>a_4= cysteinylglycine	$C_5H_9N_2O_3S$	177.0332	177.0328	1.95	0.35
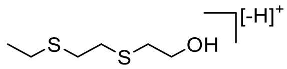<br>a_5=HETETE=neutral loss of GSH from **[M+H]**$^+$	$C_6H_{13}OS_2$	165.0403	165.0402	0.30	0.05
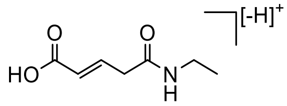	$C_7H_{10}NO_3$	156.0654	156.0655	-0.49	-0.08
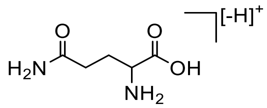	$C_5H_9N_2O_3$	145.0612	145.0608	2.84	0.41
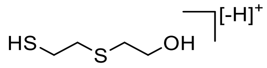<br>a_6= HETE-S	$C_4H_9OS_2$	137.0093	137.0089	2.74	0.38

Table SI 5 *(continued)*

Structure	Elemental composition	Mass (Measured)	Mass (Calculated)	Mass Error [ppm]	Mass Error [mDa]
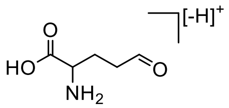	$C_5H_8NO_3$	130.0502	130.0499	2.21	0.29
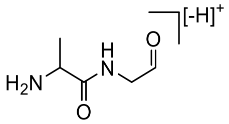	$C_5H_9N_2O_2$	129.0661	129.0659	1.74	0.22
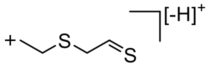	$C_4H_7S_2$	118.9985	118.9984	1.23	0.15
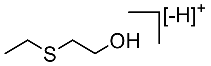	C_4H_9OS	105.0372	105.0369	3.61	0.38

a_7= HETE

Table SI 6 Product ions of single protonated GSH-ETETE-GSH

Structure	Elemental composition	Mass (Measured)	Mass (Calculated)	Mass Error [ppm]	Mass Error [mDa]
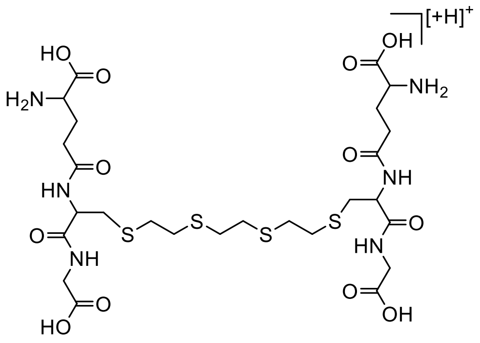 $[M+H]^+$	$C_{26}H_{45}N_6O_{12}S_4$	761.1971	761.1973	-0.25	-0.19
**$[M+H-H_2O]^+$**	$C_{26}H_{43}N_6O_{11}S_4$	743.1867	743.1867	-0.01	-0.01
**$[M+H-2H_2O]^+$**	$C_{26}H_{41}N_6O_{10}S_4$	725.1745	725.1762	-2.28	-1.66
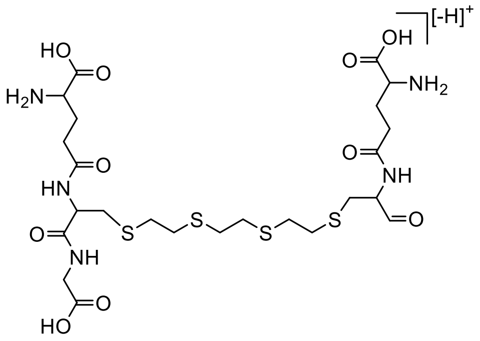 b_1= neutral loss of glycine from **$[M+H]^+$**	$C_{24}H_{40}N_5O_{10}S_4$	686.1645	686.1653	-1.06	-0.73
**$[b1-H_2O]^+$**	$C_{24}H_{38}N_5O_9S_4$	668.1536	668.1547	-1.65	-1.10
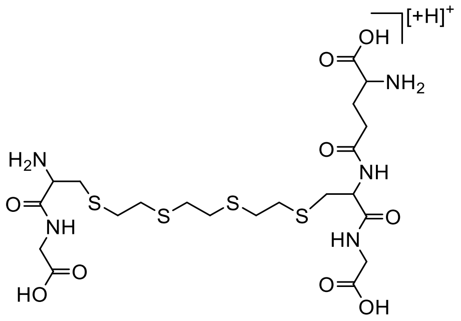 b_2= neutral loss of pyroglutamic acid from **$[M+H]^+$**	$C_{21}H_{38}N_5O_9S_4$	632.1550	632.1547	0.43	0.27
**$[b2-H_2O]^+$**	$C_{21}H_{36}N_5O_8S_4$	614.1445	614.1441	0.63	0.39

Table SI 6 *(continued)*

Structure	Elemental composition	Mass (Measured)	Mass (Calculated)	Mass Error [ppm]	Mass Error [mDa]
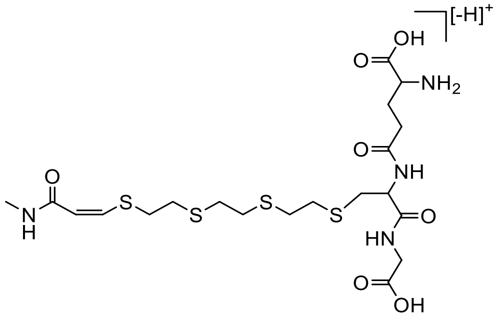	$C_{20}H_{33}N_4O_7S_4$	569.1221	569.1227	-1.07	-0.61
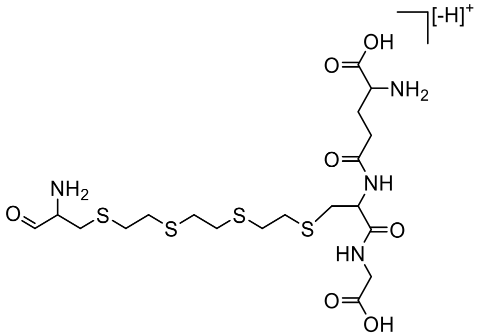 b$_3$= neutral loss of pyroglutamic acid and glycine from **[M+H]$^+$**	$C_{19}H_{33}N_4O_7S_4$	557.1227	557.1227	0.03	0.01
**[b3-H$_2$O]$^+$**	$C_{19}H_{31}N_4O_6S_4$	539.1108	539.1121	-2.34	-1.26
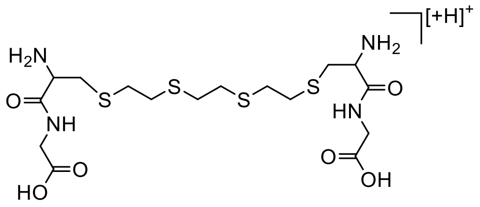 b$_4$= neutral loss of two pyroglutamic acid from **[M+H]$^+$**	$C_{16}H_{31}N_4O_6S_4$	503.1124	503.1121	0.69	0.34
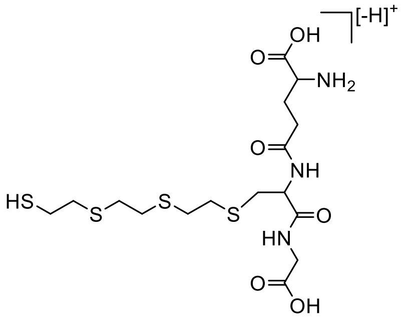 b$_5$= neutral loss of ɣ-glu-ala-glc from **[M+H]$^+$**	$C_{16}H_{28}N_3O_6S_4$	486.0859	486.0855	0.78	0.38

Table SI 6 *(continued)*

Structure	Elemental composition	Mass (Measured)	Mass (Calculated)	Mass Error [ppm]	Mass Error [mDa]
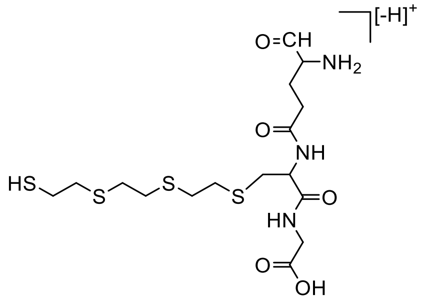	$C_{16}H_{28}N_3O_5S_4$	470.0911	470.0906	1.06	0.50
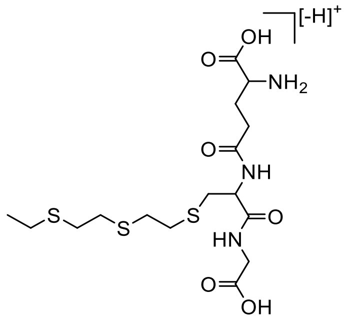 b$_6$= neutral loss of GSH from **[M+H]$^+$**	$C_{16}H_{28}N_3O_5S_3$	454.1137	454.1135	0.49	0.22
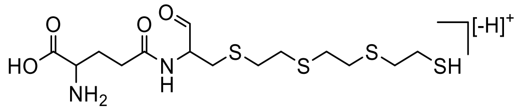	$C_{14}H_{25}N_2O_4S_4$	413.0689	413.0692	-0.65	-0.27
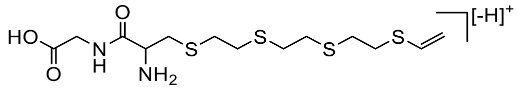	$C_{13}H_{23}N_2O_3S_4$	383.0588	383.0586	0.44	0.17
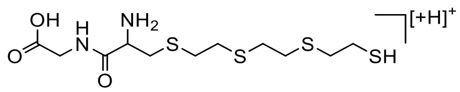	$C_{11}H_{23}N_2O_3S_4$	359.0593	359.0586	1.91	0.69
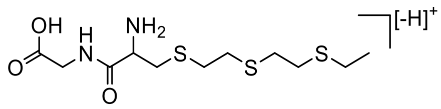	$C_{11}H_{21}N_2O_3S_3$	325.0714	325.0709	1.60	0.52
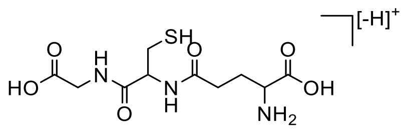	$C_{10}H_{16}N_3O_6S$	306.0756	306.0754	0.55	0.17

Table SI 6 *(continued)*

Structure	Elemental composition	Mass (Measured)	Mass (Calculated)	Mass Error [ppm]	Mass Error [mDa]
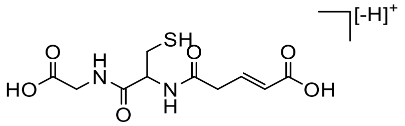	$C_{10}H_{13}N_2O_6S$	289.0487	289.0489	-0.74	-0.21
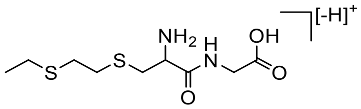	$C_9H_{17}N_2O_3S_2$	265.0688	265.0675	4.73	1.25
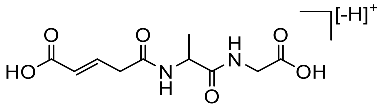	$C_{10}H_{13}N_2O_6$	257.0772	257.0768	1.65	0.42
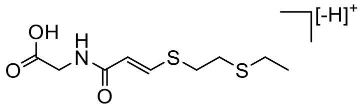	$C_9H_{14}NO_3S_2$	248.0409	248.0410	-0.22	-0.05
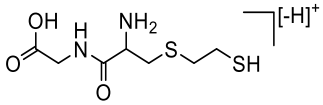	$C_7H_{13}N_2O_3S_2$	237.0367	237.0362	2.00	0.47
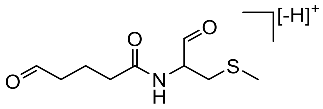	$C_9H_{14}NO_3S$	216.0680	216.0689	-3.92	-0.85
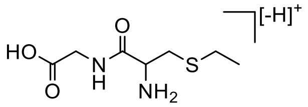	$C_7H_{13}N_2O_3S$	205.0645	205.0641	1.66	0.34
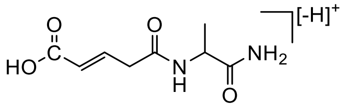	$C_8H_{11}N_2O_4$	199.0710	199.0713	-1.53	-0.30
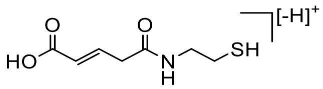	$C_7H_{10}NO_3S$	188.0379	188.0376	1.48	0.28
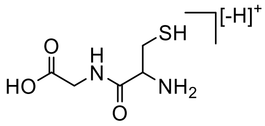 b_7= cysteinylglycine	$C_5H_9N_2O_3S$	177.0331	177.0328	1.67	0.30

Table SI 6 *(continued)*

Structure	Elemental composition	Mass (Measured)	Mass (Calculated)	Mass Error [ppm]	Mass Error [mDa]
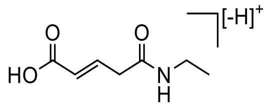	$C_7H_{10}NO_3$	156.0661	156.0655	3.59	0.56
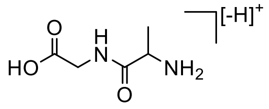	$C_5H_9N_2O_3$	145.0609	145.0608	0.90	0.13
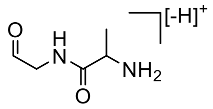	$C_5H_9N_2O_2$	129.0657	129.0659	-0.89	-0.12
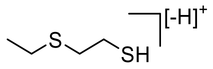	$C_4H_9S_2$	121.0142	121.0140	1.88	0.23

Table SI 7 Product ions of single protonated HETETE-Cys

Structure	Elemental composition	Mass (Measured)	Mass (Calculated)	Mass Error [ppm]	Mass Error [mDa]
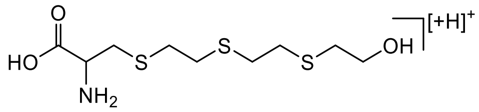 **[M+H]$^+$**	$C_9H_{20}NO_3S_3$	286.0600	286.0600	0.01	0
**[M+H-H$_2$O]$^+$**	$C_9H_{18}NO_2S_3$	268.0494	268.0494	-0.02	0
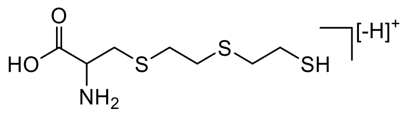	$C_7H_{14}NO_2S_3$	240.0177	240.0181	-1.62	-0.39
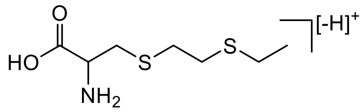	$C_7H_{14}NO_2S_2$	208.0459	208.0460	-0.52	-0.11
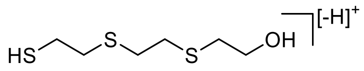 c_3= HETETE-S	$C_6H_{13}OS_3$	197.0119	197.0123	-2.07	-0.41
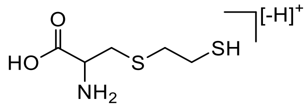	$C_5H_{10}NO_2S_2$	180.0146	180.0147	-0.69	-0.12
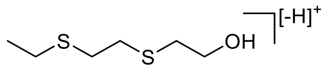 c_5= HETETE=neutral loss of cysteine from **[M+H]$^+$**	$C_6H_{13}OS_2$	165.0397	165.0402	-3.34	-0.55
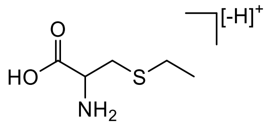	$C_5H_{10}NO_2S$	148.0426	148.0427	-0.44	-0.06
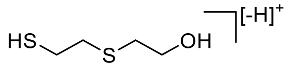 c_7= HETE-S	$C_3H_6NO_2S$	137.0091	137.0089	0.94	0.13
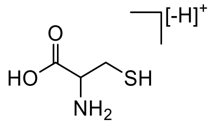	$C_4H_9OS_2$	120.0115	120.0114	1.38	0.17
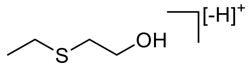 c_9= HETE	C_4H_9OS	105.0368	105.0369	-0.12	-0.01

Table SI 8 Product ions of single protonated Cys-ETETE-Cys

Structure	Elemental composition	Mass (Measured)	Mass (Calculated)	Mass Error [ppm]	Mass Error [mDa]
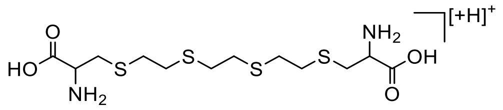 **[M+H]$^+$**	$C_{12}H_{25}N_2O_4S_4$	389.0692	389.0692	0.17	0.07
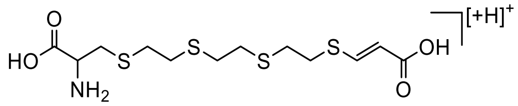	$C_{12}H_{22}NO_4S_4$	372.0421	372.0426	-1.45	-0.54
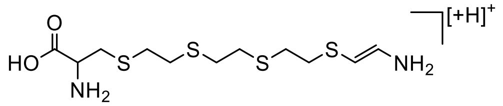	$C_{11}H_{23}N_2O_2S_4$	343.0635	343.0637	-0.45	-0.16
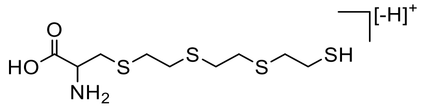	$C_9H_{18}NO_2S_4$	300.0218	300.0215	1.17	0.35
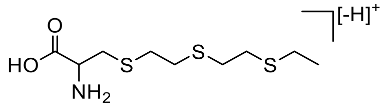 d_1= neutral loss of cysteine from **[M+H]$^+$**	$C_9H_{18}NO_2S_3$	268.0494	268.0494	-0.08	-0.02
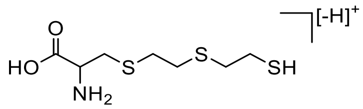	$C_7H_{14}NO_2S_3$	240.0179	240.0181	-0.92	-0.22
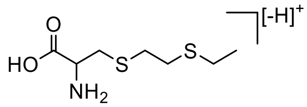	$C_7H_{14}NO_2S_2$	208.0459	208.0460	-0.93	-0.19
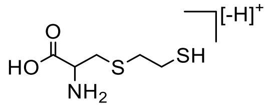	$C_5H_{10}NO_2S_2$	180.0146	180.0146	-0.78	-0.14
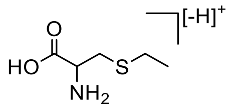	$C_5H_{10}NO_2S$	148.0427	148.0426	-0.11	-0.02
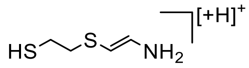	$C_4H_{10}NS_2$	136.0249	136.0249	-0.09	-0.01

Table SI 8 *(continued)*

Structure	Elemental composition	Mass (Measured)	Mass (Calculated)	Mass Error [ppm]	Mass Error [mDa]
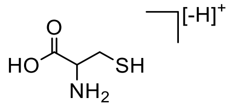	$C_3H_6NO_2S$	120.0115	120.0116	0.73	0.09
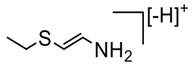	C_4H_8NS	102.0369	102.0372	-2.51	-0.26
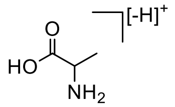	$C_3H_6NO_2$	88.0392	88.0393	-1.17	-0.1
